# Residential segregation exacerbates the effects of cardiovascular disease on cognition in Black older adults

**DOI:** 10.1093/geroni/igaf122.3839

**Published:** 2025-12-31

**Authors:** Alexandra Clark, Ashley Chikkala, Abbey Hamlin, Adam Bush, Wassim Tarraf, Alexandra Weigand, Michael Marsiske, Kelsey Thomas

**Affiliations:** University of Texas at Austin, Austin, Texas, United States; University of Texas at Austin, Austin, Texas, United States; University of Texas at Austin, Austin, Texas, United States; University of Texas at Austin, Austin, Texas, United States; Wayne State University, Detroit, Michigan, United States; University of California, San Francisco, San Francisco, California, United States; University of Florida, Gainesville, Florida, United States; University of California, San Diego, La Jolla, California, United States

## Abstract

Black older adults experience higher rates of cardiovascular disease (CVD) compared to non-Hispanic Whites, and CVD is a known risk factor for dementia. Black older adults are also more likely to reside in racially segregated neighborhoods with limited resources and access to healthcare, which may exacerbate the negative effects of CVD on cognition. The present study examined the interactive effects of CVD and segregation on memory trajectories and 10-year incidence of mild cognitive impairment (MCI) among 662 Black older adults enrolled in the Advanced Cognitive Training for Independent and Vital Elderly study. Baseline residential segregation was assessed at tract and county levels using dissimilarity and interaction indices. Blood pressure measurements (systolic ¬– diastolic) were used to calculate pulse pressure as a proxy for CVD. Outcomes included memory composites for baseline, post-intervention, 1-, 2-, 3-, 5-, and 10-year follow-ups as well as 10-year incidence of MCI. Latent growth curve models, adjusted for demographic and health factors, revealed significant segregation x CVD interactions on average memory levels (Bs≥-.48, ps≤.02), but not rates of decline. In highly segregated counties, Black older adults with elevated CVD demonstrated worse memory than those with lower CVD; in less segregated counties, memory levels did not differ by CVD. Logistic regressions revealed a similar interactive effect on 10-year incidence of MCI (OR = 1.22, p=.04); in highly segregated counties, individuals with high CVD demonstrated higher odds of MCI compared to those with low CVD. Multi-domain interventions addressing CVD risk and structural inequality may reduce dementia disparities in Black older adults.

